# Are three recovery days enough? Elevated match day creatine kinase levels under congested schedules in youth soccer

**DOI:** 10.5114/biolsport.2026.157992

**Published:** 2026-03-24

**Authors:** Gabor Schuth, Gyorgy Szigeti, Gergely Dobreff, Peter Revisnyei, Tim Gabbett, Adam Szilas, Gabor Pavlik

**Affiliations:** 1 Hungarian Football Federation, Department of Sport Medicine and Sport Science, Budapest, Hungary; 2 Hungarian University of Sports Science, Department of Health Sciences and Sport Medicine, Budapest, Hungary; 3 Budapest University of Technology and Economics (BME), Department of Telecommunications and Artificial Intelligence, Faculty of Electrical Engineering and Informatics, Budapest, Hungary; 4 Gabbett Performance Solutions, Brisbane, Queensland, Australia

**Keywords:** Adolescent Athletes, Athletic Performance, Biomarkers, Muscle Damage, Muscle Fatigue, Residual Fatigue, Recovery, Global Positioning System, Training Load

## Abstract

We examined if creatine kinase (CK) activity differed between congested (CS) and non-congested schedules (NCS) in elite youth national team soccer players. The CK activity of 188 players (16.53 ± 1.78 years) was measured in national team training camps (2489 individual data points). Training load one day before the match (MD-1) was monitored with global positioning system (GPS) devices. CS occurred, when the athlete played at least 60 minutes in the previous 3 days leading into the match (MD). CK_MD_ was significantly higher in CS than in NCS (median: 309 vs 220 U/L, p < 0.05). More recovery days between matches were associated with lower values on the second CK_MD_. CK values one (CK_MD+1_: 678 vs 487 U/L) and two days after the match (CK_MD+2_: 395 vs 303 U/L) were higher for CS (p < 0.05), however relative CK changes from MD to one (CK_MD→MD+1%_: 181 vs 135%) or two days after (CK_MD→MD+2%_: 74 vs 44%) were higher for NCS (p < 0.05). 11 out of 15 MD-1 training load parameters were significantly higher for NCS (p < 0.05). CK_MD_ was sensitive to congested schedule and the number of recovery days between matches. Therefore three recovery days between matches might be inadequate for complete recovery. Higher CK_MD_, CK_MD+1_ and CK_MD+2_ values, but lower CK_MD→MD+1%_ and CK_MD→MD+2%_ changes were found for CS than for NCS. MD-1 training load was lower in CS than in NCS, making it an unlikely cause of the elevated CK_MD_ and CK_MD+1_ values. Instead, it might be the result of residual fatigue from the last match.

## INTRODUCTION

Ongoing training and match load, as well as fatigue monitoring are crucial in modern soccer. Training monitoring is particularly relevant for national team players, who have international duties in addition to their club fixtures. One of the most widely used biomarkers to monitor muscle fatigue in soccer, is creatine kinase (CK) [1]. CK accumulates in the blood in response to training and match load, mainly as a result of eccentric muscle contractions (e.g., during decelerations and changes of direction) [2]. Many studies have used CK to follow muscle recovery after matches [3–5]. CK reaches its peak 24 hours post-activity in team sports but restoration to baseline levels may take 42–120 hours [6, 7].

Congested schedules (CS) are a hot topic in soccer as they may influence players’ subjective readiness to play, match physical, technical and tactical performance as well as injury risk [8]. According to a recent definition, CS occur when at least two consecutive matches are played with less than 96 hours of recovery [8].

There is limited evidence examining the CK response in CS and non-congested schedules (NCS) in soccer players. In a study with players from the top three divisions, the experimental group played three consecutive matches with three and four recovery days, while the control group only trained during this period [9]. Post-match CK response was highest after the second match, which was not explained by the match physical performance, but rather, the shorter (3 days) pre-match recovery time. In a more applied study, CS weeks (two matches within 4 days) and NCS weeks (two matches within 5 or more days) were compared in 23 elite adult players [10]. Match day (CK_MD_) and the following day’s (CK_MD+1_) CK did not differ between conditions, but CK two days after the match (CK_MD+2_) was significantly higher in CS. Another study compared the CK response of a Premier League team in CS (less than 4 days recovery between 2 matches) and NCS (more than 4 recovery days) during two seasons [11]. Playing at least 60 minutes in the first game resulted in significantly higher second CK_MD_ values in CS, which was confirmed by the higher subjective pre-match fatigue. Interestingly these differences between CS and NCS did not exist for CK_MD+1_.

Even fewer studies have examined the same topic in the national team context. In one study, Croatian National Team players were followed during the World Cup preparation and group stage [12]. However, only 11 players met the inclusion criteria, they did not measure CK_MD_, and five recovery days between matches was afforded to players, meaning they did not meet the definition of CS. CK dynamics of youth players during congested schedules is another gap in the scientific literature. One study did not find any differences in the CK response in young soccer players around and post-peak height velocity [13]. On the other hand, U15 players needed 48 hours to recover CK after a match, while 24 hours were sufficient for the CK activity of U13 players to return to baseline, suggesting that biological maturity might affect recovery efficiency [14]. A recent work published CK, C-reactive protein (CRP) and subjective markers during a 9-match CS of the Brazilian U-20 national team [15]. Even though they did not measure CK_MD_, post-match CK response was higher after the first four matches, when between-match recovery time was shorter (48 vs 72 hours).

Therefore, this study analysed the effect of congested schedules on CK dynamics in the days following matches. More specifically, the purposes of our study were to : 1) compare CK_MD_ values between CS and NCS as well as with different number of recovery days, 2) examine post-match (CKMD+1, CKMD+2, CKMD→MD+1%, CKMD→MD+2% change) CK response and 3) the possible effect of MD-1 training load on CK_MD_ and CK_MD+1_ values in youth national team soccer players.

## MATERIALS AND METHODS

### Subjects

One hundred and eighty-eight U15-U21 youth national team soccer players (age: 16.5 ± 1.8 years, height: 181.0 ± 6.3 cm, mass: 72.6 ± 7.2 kg) met the inclusion criteria with 2489 individual data points between March 2016 and June 2023. The athletes trained 5–7 times a week and played 1–2 matches in their club. The players (and if < 18 years) their parents gave their informed consent to take part in this study, which was approved by the Ethical Committee of the Hungarian University of Sports Science (KEB/No9/2020). The investigation conformed to the Code of Ethics of the World Medical Association [16].

### Design

**FIG. 1:**
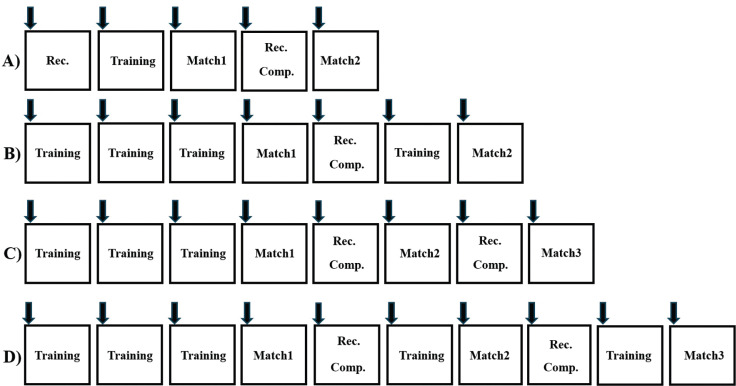
Main types of training camps of the national team preparation. A) After playing in their club at the weekend, the soccer players arrive to the national team on Sunday afternoon taking part in a recovery session. One or two tactical trainings help to prepare them for the international match on Tuesday. Players with <45 minutes playing time have a compensation session, the other players a recovery programme on Wednesday. The training camp finishes with another international friendly game on Thursday. B) Typical preparation programme of the U21 team. After 3-5 training sessions, they play an international match, which is followed by two training sessions and a second match. C) Previous youth tournaments were organized, where three friendly matches were played with one recovery day in-between. D) During European Qualifications three matches are played with two recovery days separating them. Black arrows indicate daily creatine-kinase measurements. Rec.= recovery session, comp.=compensation session.

**FIG. 2:**
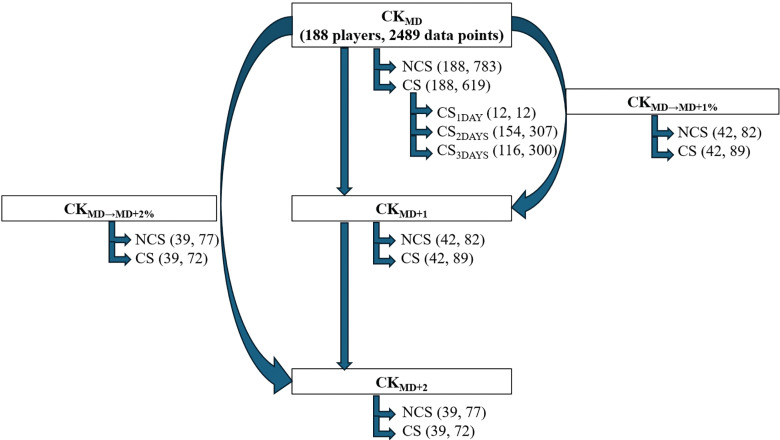
Number of players, creatine-kinase (CK) data points (in the parenthesis) according to congested (CS) and non-congested (NCS) schedule in the study. CK_MD_: CK on match day, CK_MD+1/MD+2_: CK one or two days after the match, CK_MD→MD+1/+2%_: relative CK change (%) from match day to one or two days after, CS_1/2/3DAYS_: congested schedules with 1/2/3 recovery days before the second match day. Recovery day indicates the number of days since the last match. E.g. CS (39,72) for CK_MD+2_ means that 39 players had 72 individual CK data points two days after the match. These players also had CK values on match day and one day after the match.

Players took part in different national team training camps during the study period (Figure 1). CK was measured each morning and GPS/GNSS devices were used to record movement data during training sessions and matches. The existence of match day (MD) CK data was the first inclusion criteria (Figure 2.). If any GPS data of the three days leading to MD (MD-3, MD-2, MD-1) was missing, the player was excluded from the sample (1087 data points out of 2489, Figure 2). If available, CK values on the first and second days after the match (CK_MD+1_, CK_MD+2_) were also added to the sample. Figure 2 shows the number of data points for each scenario (CK_MD_, CKMD+1, CKMD→MD+1%, CKMD+2, CKMD→MD+2%).

### Congested schedule

MD was considered as CS when the player played at least 60 minutes on one of the preceding days (MD-1, MD-2, MD-3) [11]. All other cases were treated as non-congested schedule (NCS). Therefore, four possible categories were available:

1. NCS: The player trained on MD-3, MD-2 and MD-1 or his match playing time was less than 60 minutes.
2. Congested schedule with 3 days rest (CS_3DAYS_): The athlete played at least 60 minutes on MD-3. MD-1 and MD-2 consisted of training sessions or matches with less than 60 minutes playing time.
3. Congested schedule with 2 days rest (CS_2DAYS_): The player played at least 60 minutes on MD-2. On MD-1 he trained or played less than 60 minutes.
4. Congested schedule with 1 day rest (CS_1DAY_): MD-1 contained a match with at least 60 minutes playing time.

### Methodology Creatine kinase

CK samples were taken each morning from fingertips between 8–10 am after overnight fasting as described previously [17, 18]. Whole capillary blood was analysed using Reflotron Plus Clinical Chemistry Analyser (Roche Diagnostics) between 2016 and 2021. The validity [19] and reliability [20] of this system has been shown previously. Between 2022–2023, SimplexTas 101 Immuno Chemistry System (Tascom, South Korea) was used to determine CK from capillary blood. The validity of the system was checked against the output of an accredited medical laboratory using venous samples [21]. Based on our previous comparison, a correction equation was applied between the Reflotron and Tascom systems, which allowed us to merge the whole CK dataset and increase sample size [22]. The CK results provided by the two machines were significantly correlated (r = 0.92, p < 0.05) [22], the mean absolute difference between the two machines was 110 U/L (95% confidence intervals: 94–127 U/L). The mean relative difference between machines was 56.5% (95% confidence intervals: 45–68%).

### External load monitoring

The training and match activity profile of the players were monitored with 10 Hz GPS/GNSS units (Catapult S5 for field players and G5 for goalkeepers between 2016- June 2019, Catapult S7 for field players and G7 for goalkeepers from July 2019, Catapultsports, Australia). The players wore the units in custom-made vests between the shoulder blades, which minimized the movements of the units but did not limit the rotation of the torso. The validity of the Catapult S5 [23] and S7 [24] units has been published in previous studies. To quantify GPS signal quality, horizontal dilution of precision (HDOP) was applied; we only included activities when the average HDOP of the periods was < 1.1 [25]. The units also contained microsensors (accelerometer, gyroscope, magnetometer) with 100 Hz sampling frequency; their validity has been shown previously [26]. The 15 GPS/microsensor-derived parameters used in this study are defined in Table 1.

**TABLE 1:** Parameter groups, parameter names and parameter definitions (thresholds) used to characterize the training load one day before the match (MD-1).

Parameter group	Parameter name	Definition
Global Positioning System	Total distance	Distance covered in all velocity zones (m)
	High-intensity distance	Distance above 19.8 km/h (m)
	Sprint distance	Distance above 25.1 km/h (m)
	Distance >30 km/h	Distance above 30 km/h (m)
	Maximum velocity	Maximum velocity during a training session or match(km/h)
	High metabolic power distance	Distance above 25.5 W/kg (m)
	Number of accelerations	Number of accelerations >2m/s^2^
	Number of decelerations	Number of decelerations >-2m/s^2^

Inertial Movement Analysis (IMA)	Number of IMA Events	Number of accelerations, decelerations, left and right changes of directions above 1.5 m/s
	Number of high-intensity IMA Events	Number of accelerations, decelerations, left and right changes of directions above 3.5 m/s
	Number of high-intensity IMA accelerations	Number of IMA-based accelerations above 3.5 m/s
	Number of high-intensity IMA decelerations	Number of IMA-based decelerations above 3.5 m/s
	Number of high-intensity IMA left changes of directions	Number of IMA-based left changes of directions above 3.5 m/s
	Number of high-intensity IMA right changes of directions	Number of IMA-based right changes of directions above 3.5 m/s
	Player Load™	Total Player Load (AU)

Note: Parameters are based on Global Positioning System (GPS) data and Inertial Movement Analysis (IMA). The main difference is that the latter does not require GPS signal. MD-1 training load was analysed whether it differs between congested and non-congested schedules and influences post-match creatine kinase response.

### Statistical Analysis

Normality was checked with the Shapiro-Wilk test (p < 0.05) for each CK and GPS parameter distributions. If normality was violated, median and percentiles (2.5/5/10/90/95/97.5) were used as descriptive statistics. To compare two non-normally distributed samples, the Mann-Whitney-U test was used (significance set at p < 0.05). CKMD, CKMD+1, CKMD+2, CKMD→MD+1% as well as CKMD→MD+2% for CS and NCS, MD-1 training sessions’ GPS data for CS and NCS were analysed using Mann-Whitney-U tests. In these cases, U and p values, Z statistics and r effect sizes were reported to characterize the difference between samples. Effect sizes < 0.3 were rated as small, between 0.3–0.5 as medium effect and > 0.5 as large effect. To compare more than three, non-normally distributed samples, Kruskal-Wallis ANOVA was used (significance set at p < 0.05). In case of significant results, Mann-Whitney-U tests were used as post-hoc tests to compare the groups. CK_MD_ values with different recovery days (NCS, CS_3DAYS_, CS_2DAYS_, CS_1DAY_) were analysed this way. All statistical analyses were performed in Python (version 3.10.6) using Sklearn (version 1.1.2) and Statsmodels (version 0.13.2).

## RESULTS

### CK_MD_ and the effect of congested schedule

CK_MD_ median and percentiles are shown for CS and NCS, as well as for CS with different recovery days in Table 2. CK_MD_ for CS and NCS, as well as for CS with 2 or 3 recovery days were not normally distributed (p < 0.01). The only exception was CK_MD_ CS_1DAY_ (p > 0.05), but due to the low sample size (n = 12), non-parametric statistical procedures were applied. CK_MD_ was significantly higher for CS than for NCS (p < 0.01). Significant differences were found between MD CK values based on the recovery days since the last match (χ^2^ = 125.05, p < 0.01). According to the post-hoc tests, CS_1DAY_ CK_MD_ was significantly higher than CS_3DAYS_ CK_MD_ (U = 2613, Z = 2.65, p < 0.01, r = 0.15) and NCS (U = 7333.5, Z = 3.34, p < 0.01, r = 0.12). Furthermore, CS_2DAYS_ CK_MD_ was significantly higher than CS_3DAYS_ CK_MD_ (U = 58653.5, Z = 5.83, p < 0.01, r = 0.24) and NCS (U = 169686, Z = 10.59, p < 0.01, r = 0.32). Finally, CS_3DAYS_ CK_MD_ was significantly higher than NCS (U = 137745, Z = 4.41, p < 0.01, r = 0.13).

**TABLE 2:** Match day (MD) creatine-kinase percentiles for all data points, as well as for congested and non-congested schedules.

Data set	Congested schedule	Number of recovery days	Number of data points	2.5	5	10	Median	90	95	97.5
MD	-	-	2489	63	82	112	242	519	630	766

MD	No	-	783	50	69	99	220	475	579	669
	Yes	-	619	85	108	141	309	617	733	854

MD	No	-	783	50	69	99	220	475	579	669
	Yes	Three	300	73	108	131	263	534	619	741
	Yes	Two	307	93	120	157	355	670	775	932
	Yes	One	12	139	170	222	493	839	901	923

Note: Data points for congested schedules are further categorized based on the number of recovery days (number of days since the last match). Number of data points in each subset are also shown.

### CK_MD+1_ and CK_MD→MD+1%_ CK change

**FIG. 3:**
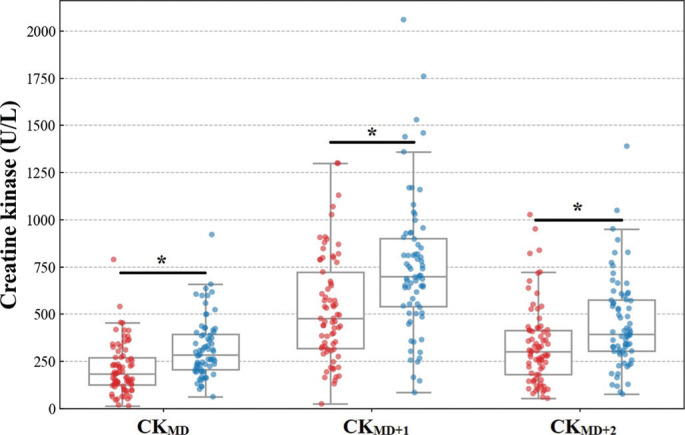
Absolute creatine-kinase (CK) values for match day (CK_MD_), one (CK_MD+1_) and two days after the match (CK_MD+2_) for noncongested (blue) and congested (red) schedules. * Indicates a significant difference according to the Mann-Whitney-U test between congested and non-congested schedules for that day (match day, one or two days after the match). Only players with CK_MD_, CK_MD+1_ and CK_MD+2_ values are shown on the figure. The line inside the boxes indicates the median value, the length of the box represents the first and third quartiles of the data points (the length of the box is the interquartile range). The whiskers (lines) outside the box are 1.5 times interquartile ranges from the box. The coloured dots represent the individual data points.

CK_MD+1_ for CS and NCS as well as CK_MD→MD+1%_ for CS and NCS were not normally distributed (p < 0.05). CK_MD+1_ was significantly higher with small effect size in CS than in NCS (median: 678 vs 487 U/L, U = 4678, Z = 3.18, p < 0.01, r = 0.24) (Figure 3, Table 3). On the contrary, CK_MD→MD+1%_ was significantly higher with small effect size in NCS than in CS (median: 181 vs 135%, U = 2755, Z = -2.76, p < 0.01, r = 0.21) (Table 3).

**TABLE 3:** Creatine-kinase (CK) percentiles for match day (CK_MD_), one (CK_MD+1_) and two days after the match (CK_MD+2_) as well as percentage CK change from match day to the day after (CK_MD→MD+1%_) and two days after the match (CK_MD→MD+2%_) for congested and non-congested schedules.

Data subset	Parameter	Congested schedule	Number of data points	2.5	5	10	Median	90	95	97.5
MD+1	CKMD	No	82	46	52	65	183	360	413	453
		Yes	89	69	81	120	287	559	613	655
	CKMD+1	No	82	149	167	214	487	906	1028	1129
		Yes	89	151	173	266	678	1162	1408	1516
	CKMD MD+1%	No	82	−23	4	34	181	331	592	772
		Yes	89	−10	3	21	135	285	317	366

MD+2	CKMD	No	77	43	51	64	183	384	426	464
		Yes	72	113	126	160	285	556	612	642
	CKMD+1	No	73	145	166	209	478	894	1045	1164
		Yes	72	162	253	309	701	1169	1449	1582
	CKMD+2	No	77	78	94	105	303	622	744	850
		Yes	72	112	128	189	395	753	858	974
	CKMD MD+2%	No	77	−59	−37	−32	74	198	239	332
		Yes	72	−53	−36	−22	44	106	142	250

Note: For MD+1 subset, players were required to have CK_MD_ and CK_MD+1_ values, for MD+2 subset CK_MD_, CK_MD+1_ and CK_MD+2_ CK values. This is the reason why number of data points and percentiles are different for the MD+1 and MD+2 subsets. E.g. the first row shows the MD CK percentiles for non-congested schedules for the players, who had CK_MD_ and CK_MD+1_ values.

### CK_MD+2_ and CK_MD→MD+2%_ CK change

CK_MD+2_ for CS and NCS as well as CK_MD→MD+2%_ for CS and NCS were not normally distributed (p < 0.05). CK_MD+2_ was significantly higher with small effect size in CS than in NCS (median: 395 vs 303 U/L, U = 3663.5, Z = 3.39, p < 0.01, r = 0.28) (Figure 3, Table 3). On the contrary, CK_MD→MD+2%_ was significantly higher with small effect size in NCS than in CS (median: 74 vs 44%, U = 2156, Z = -2.34, p < 0.05, r = 0.19) (Table 3).

### MD-1 training GPS data and the effect of congested schedule

MD-1 training GPS parameters for CS and NCS were not normally distributed (p < 0.01) (Table 4). Table 4 shows the percentile values of different MD-1 training load parameters for CS and NCS. The number of data points for CS and NCS differs between parameters. This is caused by the fact that due to poor GPS signal quality (HDOP < 1.1), GPS-based parameters were excluded from the analysis in some cases, which did not influence IMA-derived parameters. MD-1 training load parameters for NCS were significantly higher with small effect size than those for CS, except from sprint distance, distance > 30 km/h, maximal velocity and number of accelerations > 2 m/s^2^ (Table 5).

**TABLE 4:** Percentiles of training load data one day before the match (MD-1) for non-congested (CS no) and congested schedules (CS yes).

Parameter	Congested schedule	2.5	5	10	Median	90	95	97.5	Number of data points	Number of players	Shapiro p
Total Player Load (AU)	No	79	145	212	384	499	540	564	530	142	<0.001
	Yes	39	50	124	363	489	532	581	234	104	<0.001
Total Distance (m)	No	1397	1815	2115	3687	4732	5154	5560	491	140	<0.001
	Yes	475	960	1446	3546	4682	5038	5285	212	99	<0.001
High-intensity distance (m)	No	0	0	10	92	236	265	311	491	140	<0.001
	Yes	0	0	0	74	207	246	261	212	99	<0.001
Sprint distance (m)	No	0	0	0	5	44	70	78	491	140	<0.001
	Yes	0	0	0	0	40	57	77	212	99	<0.001
Distance >30 km/h (m)	No	0	0	0	0	0	0	7	491	140	<0.001
	Yes	0	0	0	0	0	1	9	212	99	<0.001
Maximal velocity (km/h)	No	18.56	19.37	21.45	25.61	28.92	29.61	30.23	491	140	<0.001
	Yes	12.49	16.96	18.72	25.15	29.27	29.89	30.55	212	99	<0.001
High metabolic power distance (m)	No	64	78	124	379	605	667	707	491	140	<0.001
	Yes	2	23	63	304	563	605	677	212	99	<0.05
Total number of micromovements	No	15	72	142	263	396	431	477	530	142	<0.001
	Yes	0	2	42	220	377	442	481	234	104	<0.001
Number of high-intensity micromovements	No	0.2	2.0	3.0	12.0	24.0	29.0	37.3	530	142	<0.001
	Yes	0	0	1.0	8.0	23.0	31.1	38.7	234	104	<0.001
Number of high-intensity accelerations	No	0	0	1.0	3.0	8.0	10.0	11.8	530	142	<0.001
	Yes	0	0	0	3.0	7.0	11.0	13.0	234	104	<0.001
Number of high-intensity decelerations	No	0	0	0	2.0	6.0	8.5	10.0	530	142	<0.001
	Yes	0	0	0	1.0	6.0	7.0	13.0	234	104	<0.001
Number of high-intensity left changes of directions	No	0	0	0	2.0	6.0	8.0	10.0	530	142	<0.001
	Yes	0	0	0	2.0	5.0	9.0	10.2	234	104	<0.001
Number of high-intensity right changes of directions	No	0	0	0	3.0	8.0	9.0	11.0	530	142	<0.001
	Yes	0	0	0	2.0	6.0	7.0	10.2	234	104	<0.001
Number of accelerations	No	0	0	0	7.0	19.0	22.6	26.8	530	142	<0.001
	Yes	0	0	0	5.0	16.0	19.0	21.2	234	104	<0.001
Number of decelerations	No	0	0	0	7.0	17.1	21.0	22.0	530	142	<0.001
	Yes	0	0	0	4.0	14.0	18.0	21.0	234	104	<0.001

Note: Both Global Positioning System (GPS) and Inertial Movement Analysis (IMA)-based metrics are shown. Every two rows show the same MD-1 training load parameter for non-congested (upper) and congested schedules (lower row). Shapiro (p)= p value of the Shapiro-Wilk normality test for each parameter for non-congested and congested schedules. High-intensity distance (>19.8 km/h), sprint distance (>25.1 km/h), high metabolic power distance (>25.5 W/kg), high-intensity micromovements/accelerations/decelerations/left changes of directions/right changes of directions (>3.5 m/s, microsensor based), number of accelerations/decelerations (>± 2 m/s^2^, GPS-based).

**TABLE 5:** The results of the Mann-Whitney U statistical tests comparing training load one day before the match for congested (CS) and non-congested (NCS) schedules.

U value	p value	Z value	r effect size	Parameter
52138	0.000	−3.511	0.127	Total Player Load (AU)
47072	0.044	−2.013	0.076	Total Distance (m)
45474	0.008	−2.660	0.100	High-intensity distance (m)
48561	0.140	−1.410	0.053	Sprint distance (m)
52531	0.587	0.196	0.007	Distance >30 km/h (m)
47571	0.070	−1.811	0.068	Maximal velocity (km/h)
42661	0.000	−3.798	0.143	High metabolic power distance (m)
48740	0.000	−4.719	0.171	Total number of micromovements
48268	0.000	−4.887	0.177	Number of high-intensity micromovements
53214	0.002	−3.128	0.113	Number of high-intensity accelerations
52494	0.001	−3.384	0.122	Number of high-intensity decelerations
51452	0.000	−3.755	0.136	Number of high-intensity left changes of directions
50140	0.000	−4.221	0.153	Number of high-intensity right changes of directions
56736	0.059	−1.876	0.068	Number of accelerations
55251	0.015	−2.404	0.087	Number of decelerations

Note: U= Mann-Whitney U value, p= significance (<0.05), Z statistics, r=effect size. Negative Z-value indicates that MD-1 training load for that parameter was higher for NCS than for CS. Significant differences between CS and NCS are highlighted in red. High-intensity distance (>19.8 km/h), sprint distance (>25.1 km/h), high metabolic power distance (>25.5 W/kg), high-intensity micromovements/accelerations/decelerations/left changes of directions/right changes of directions (>3.5 m/s, microsensor based), number of accelerations/decelerations (>± 2 m/s^2^, GPS-based).

## DISCUSSION

We examined the sensitivity of CK to CS in youth national team soccer players. The main findings were: 1) with fewer recovery days since the last match, CK on the second match day increased significantly, 2) CK_MD+1_ and CK_MD+2_ absolute values were significantly higher in CS than in NCS, 3) CK_MD→MD+1%_ and CK_MD→MD+2%_ relative changes were significantly higher in NCS than in CS, 4) finally most of MD-1 training GPS data were significantly higher in NCS.

CK_MD_ was significantly higher in CS (CS: 309 U/L, NCS: 220 U/L), if the player played at least 60 minutes in one of the last 3 days leading to the second match. This finding is in agreement with a recent study comparing single- and multi-match weeks in the Premier League [11]. Another study partly confirms this result: 1–3^rd^ division players of the experimental group played three consecutive matches with three and four recovery days in-between, whereas the control group only trained [9]. CK_MD_ was significantly higher for the experimental group on the second and third MD, suggesting an incomplete recovery. Another study did not find any difference in CK_MD_ between CS and NCS in elite adult soccer players [10]. These studies were executed in club environments. As far as we know, only three studies have examined the CK response to matches of national team soccer players, but direct comparison is not possible due to methodological differences. One study examined the 2014 World Cup preparation and group stage of the Croatian National team [12]. Low sample size, five recovery days between matches and lack of CK_MD_ measurement are the main limitations for comparison. Similarly, CK_MD_ was not determined for the Brazilian U-20 national team during a 9 match-long congested schedule [15]. Finally, only CK_MD+2_ values were reported for 68 adult German National team players during European Championships and World Cups over a 10-year period [19].

To the best of our knowledge, this is the first study to examine the effect of recovery time between two consecutive matches on the second CK_MD_ value. CK values were higher on the second MD when the previous match was more than three, three, two or one day before (NCS: 220 U/L, CS_3DAYS_: 263 U/L, CS_2DAYS_: 355 U/L, CS_1DAY_: 493 U/L). This confirms the sensitivity of MD CK for the recovery time and therefore residual fatigue since the last match. This finding has important practical applications, as it suggests that 72 hours between youth matches might not be enough for full recovery when the player played more than 60 minutes during the first game. This finding should be taken into consideration when organising youth soccer tournaments. Equally, coaches should consider using squad rotations effectively to manage the load of the players. Our study did not examine the mechanism/s behind the CK increase. According to a recent review, physiological fatigue (due to impaired calcium release and uptake) and mechanical fatigue (tissue damage caused by mechanical stress) might overlap [27]. These phenomena might be part of a continuum, with physiological fatigue as a starting point and muscle injury as an end point [27]. Both can elevate CK and due to its large molecule size, once entered into the bloodstream, clearance is slow [27]. Further studies should examine whether elevated CK levels on the second match day impairs match physical performance or increases injury risk during congested schedules in youth soccer players.

CK_MD+1_ values were significantly higher in CS than in NCS (median: 678 vs 487 U/L). Direct comparison with the literature is challenging, as some studies did not report exact CK values [9]. Similar CK_MD+1_ values (506 ± 242 U/L) were reported for the Croatian national team with 5 recovery days between matches as for NCS in our study [12]. However, much lower (CS: 301 U/L, NCS: 295 U/L) [10] and slightly higher values (CS: 704 U/L, NCS: 686 U/L) [11] were also published in elite adult soccer players. In contrast to our findings, these researchers did not report significant difference in the CK_MD+1_ values between CS and NCS [10, 11]. One explanation for the discrepant findings might be the different definitions for CS between studies: two matches within four [10] or five days [11] or 2–4 days in the current study.

In contrast to CK_MD+1_, CK_MD→MD+1%_ was significantly higher in NCS than in CS (NCS: 181%, CS: 135%). In case of CS, a higher CK_MD_ (CS: 287, NCS: 183 U/L) was associated with a lower relative change in CK_MD+1_ than in NCS. Similar magnitude CK_MD→MD+1%_ (NCS: 145%, CS: 116%) was reported for adult Premier League soccer players [11], but another study reported much lower relative changes (NCS: 61%, CS: 36%) [10]. Taking the timing of the measurement into consideration this finding is somewhat surprising. Similarly to our protocol, the first study measured CK 12–19.5 hours after the end of the match depending on kick off time [11]. As CK is reported to peak 24 hours after the match in team sports [5] and the authors of the second study measured CK after 24 hours [10], they expected to observe higher changes, but this was not the case. They hypothesized that this result was due to most matches occurring after the beginning of the preseason, when players were the most physically prepared [10]. Further studies either did not report exact CK_MD_ and CK_MD+1_ values [9] or did not measure CK_MD_ [12, 15], therefore cannot be used for comparison.

As CK_MD→MD+1_ absolute CK change did not differ between conditions (CS: 324, NCS: 298 U/), the influence of the repeated bout effect (RBE) might have been minimal on our results. According to the RBE, a similar magnitude second eccentric load within days or weeks will result in an attenuated muscle damage response than the first one [28, 29]. However, the short time (1–3 days) between matches might have offered inadequate recovery time for the second match, as suggested by the higher CK_MD_ values in CS.

CK_MD+2_ values were significantly higher in CS (CS: 395 U/L, NCS: 303 U/L), while CK_MD→MD+2%_ was significantly higher in NCS (NCS: 74%, CS: 44%). This suggests that our players were still recovering two days after the match, as median CK_MD+2_ was still higher than CK_MD_. This is in contrast to previous findings, where CK_MD+2_ values were lower than MD [10] or MD-1 values [12]. Unlike our population of youth players, the above findings were from studies of adult players [10, 12]. Our inclusion criteria was to include players with full matches only, whereas previous studies used either 75 minutes playing time as criteria [10] or no criteria at all [12]. Finally, we measured CK_MD+2_ ~39 hours after the end of the match compared to 48 hours [10], which might have allowed extra recovery time for the players.

Regarding the CK trend, our findings are in agreement with the literature: CK reached its peak one day after the match (measured ~15 hours after the end of it), which is within the range previously suggested [5, 10, 12]. The return of CK to baseline was outside of the scope of this study, as it is suggested to occur 42–48 [5, 10, 12] or even 72–120 hours [6, 7] after the match, and our last measurement point was ~39 hours after the final whistle.

Previous reviews have suggested that CK reaches its peak 14–48 hours after matches [5]. We found significantly higher CK_MD_ and CK_MD+1_ absolute values in CS than in NCS. Therefore, the question arises whether MD-1 training load might have influenced this trend. The results showed that MD-1 training load was significantly lower during CS (11 out of 15 GPS parameters, Table 5). This suggests that the elevated CK on MD and MD+1 were not the consequence of higher training loads on MD-1 but the residual fatigue from the previous match played within 3 days. We did not find significant differences between CS and NCS in four GPS parameters: in addition to maximal velocity, the low volume of sprint distance, distance > 30 km/h, as well as number of accelerations > 2 m/s^2^ which might explain these findings. Interestingly, most previous studies investigating CS did not examine the effect of MD-1 training load on the CK response after matches [11, 12, 15], although the studies that have been performed are equivocal. Training load of the experimental and control group did not differ statistically when playing three consecutive matches with three and four recovery days in-between [9]. These authors examined total and high-intensity distance, as well as average and maximal heart rate as training load indicators. In this case, MD-1 training load did not seem to influence post-match CK response. Conversely, MD-1 training load (i.e., relative distance, relative high-intensity distance and number of accelerations) was significantly higher in CS than in NCS in another study [10]. The authors hypothesized that decreased load on MD-2 might explain these findings. Even though MD-1 training load was higher in CS, in contrast to our findings, CK_MD_ and CK_MD+1_ did not differ significantly between CS and NCS. The elite adult population and attenuated post-match CK response might explain these discrepancies.

### Limitations

Our study has several limitations. First, several age groups (U15-U21) were included in the study to increase sample size. Biological maturity has been shown to influence CK response [14], but it should have limited effect in our sample as most players were post peak height velocity. Second, regarding the statistical methods, it should be emphasized that CS and NCS samples were not fully independent, as each player needed to have at least one CS and one NCS data point to be included in the analysis. As a result, the number of CS and NCS data points for a player are not necessarily the same, and as such, we treated the samples as independent. Third, the CK analyser was changed to another brand during the study period and GPS/GNSS was updated to a newer model of the same brand. As validation studies confirmed the validity of all of the equipment, these changes might have had limited impact on our results. Fourth, our standard recovery protocol after matches might have influenced our results as suggested by the literature [30], however it was standardized between age groups, therefore its effect should be comparable. Fifth, due to the applied nature of the study, we were not able to measure CK exactly 24 and 48 hours after the matches, which might have influenced peak CK and CK recovery kinetics [5]. Sixth, caution is needed when translating these results to a club environment, as the focus of physical preparation in the national team is to maximize player readiness for consecutive matches played with 1–3 recovery days. Finally, CK only shows one aspect of the recovery process, therefore further studies are needed to examine additional possible recovery markers (e.g. lactate-dehydrogenase, myoglobin, perceived wellbeing, etc.).

## CONCLUSIONS

These findings show the sensitivity of CK to congested schedules and its applicability to monitor muscle recovery in similar scenarios. Consistent with results from senior players, CK_MD_, CK_MD+1_ and CK_MD+2_ values were significantly higher in CS than in NCS in elite youth national team soccer players. Furthermore, as the number of recovery days between two matches decreased, the second CK_MD_ increased, suggesting an incomplete recovery 24–72 hours after the first match. This might have important implications for the scheduling of youth tournaments, where often one or two recovery days are only available between consecutive matches. Future studies should investigate whether elevated CK_MD_ values have a negative effect on match physical performance or match injury risk. As MD-1 training load was higher in NCS than in CS, it does not explain the higher postmatch CK response in CS. Rather it might be the consequence of residual fatigue from playing at least 60 minutes during the previous match. As post-match CK values were higher in CS, but absolute CK changes similar between CS and NCS, the repeated bout effect in our sample is likely to be negligible.
